# Ag-Decorated Si Microspheres Produced by Laser Ablation in Liquid: All-in-One Temperature-Feedback SERS-Based Platform for Nanosensing

**DOI:** 10.3390/ma15228091

**Published:** 2022-11-15

**Authors:** Stanislav Gurbatov, Vladislav Puzikov, Evgeny Modin, Alexander Shevlyagin, Andrey Gerasimenko, Eugeny Mitsai, Sergei A. Kulinich, Aleksandr Kuchmizhak

**Affiliations:** 1 Institute of Automation and Control Processes, Far Eastern Branch, Russian Academy of Science, 5 Radio Str., 690041 Vladivostok, Russia; 2 Far Eastern Federal University, 690041 Vladivostok, Russia; 3 CIC NanoGUNE BRTA, 20018 Donostia-San Sebastian, Spain; 4 Institute of Chemistry, Far Eastern Branch, Russian Academy of Science, 690022 Vladivostok, Russia; 5 Research Institute of Science & Technology, Tokai University, Hiratsuka 259-1292, Kanagawa, Japan

**Keywords:** pulsed laser ablation in liquid, hybrid nanomaterials, silver, silicon, plasmonics, SERS

## Abstract

Combination of dissimilar materials such as noble metals and common semiconductors within unified nanomaterials holds promise for optoelectronics, catalysis and optical sensing. Meanwhile, difficulty of obtaining such hybrid nanomaterials using common lithography-based techniques stimulates an active search for advanced, inexpensive, and straightforward fabrication methods. Here, we report one-pot one-step synthesis of Ag-decorated Si microspheres via nanosecond laser ablation of monocrystalline silicon in isopropanol containing AgNO_3_. Laser ablation of bulk silicon creates the suspension of the Si microspheres that host further preferential growth of Ag nanoclusters on their surface upon thermal-induced decomposition of AgNO_3_ species by subsequently incident laser pulses. The amount of the AgNO_3_ in the working solution controls the density, morphology, and arrangement of the Ag nanoclusters allowing them to achieve strong and uniform decoration of the Si microsphere surface. Such unique morphology makes Ag-decorated Si microspheres promising for molecular identification based on the surface-enhanced Raman scattering (SERS) effect. In particular, the designed single-particles sensing platform was shown to offer temperature-feedback modality as well as SERS signal enhancement up to 10^6^, allowing reliable detection of the adsorbed molecules and tracing their plasmon-driven catalytic transformations. Considering the ability to control the decoration degree of Si microspheres by Ag nanoclusters via amount of the AgNO_3_, the developed one-pot easy-to-implement PLAL synthesis holds promise for gram-scale production of high-quality hybrid nanomaterial for various nanophotonics and sensing applications.

## 1. Introduction

Noble-metal nanoparticles supporting optically induced resonance oscillations of their electron density (surface plasmons), as well as submicron structures made of low-loss highly refractive-index semiconductors supporting geometric Mie modes, have been the mainstream during last decades of both fundamental and applied research in the fields of nanophotonics, plasmonics, optoelectronics, catalyst, and optical sensing [1,2]. Progressive popularity of such functional nanomaterials is associated with their unique optical properties. In particular, surface plasmons offer strong light localization and enhancement at nanoscale volumes, while Mie modes permit to control radiation directivity and confine light within the related all-dielectric nanostructure boosting its inherent linear and nonlinear optical responses. Merging plasmonic and all-dielectric concepts within the unified hybrid nanostructures can be considered as a new step towards design of the advanced materials with expanded functionality [3,4]. In particular, plasmon-mediated highly energetic (also referred to as “hot”) electrons can be injected to semiconductor to enhance its light-emitting and light-absorbing properties, allowing it to realize advanced photodetectors and nanoscale coherent light emitters [5,6,7]. Moreover, combination of noble metals and semiconductors holds promise for next-generation catalyst [8] as well as realization of multi-functional optical sensing platforms benefiting from plasmon-mediated enhancement of molecular fingerprint signals via surface–enhanced photoluminescence and surface-enhanced Raman scattering (SERS) [9] and chemical inertness of all-dielectric nanostructures [10]. Moreover, monocrystalline semiconductors usually exhibit inherent characteristic Raman scattering that can be enhanced by Mie resonances [11] and can be used for local nanothermometry [12]. However, it should be noted that the optical-range resonant response for plasmonic and all-dielectric nanostructures is usually observed for plasmonic and all-dielectric nanostructures with a rather dissimilar size. This issue makes application of common multi-step lithography-based nanofabrication techniques for hybrid nanostructure formation challenging and money-consuming, stimulating the search for more straightforward, productive, and versatile fabrication approaches.

Pulsed laser ablation in liquids (PLAL) can provide an easy-to-realize and elegant way to tackle the problem of formation of hybrid metal-semiconductor nanostructures [13,14,15]. In particular, popular combinations of metals and semiconductors mixed within the functional nanomaterials via PLAL synthesis include Ag- (Au- or Pt-) decorated TiO_2_ [16,17,18,19,20] or Au-decorated Si [21,22,23,24,25,26]. These hybrid nanomaterials proved their usefulness for optical and gas sensing, catalysis, and nanophotonics, justifying PLAP as advanced and versatile fabrication technology. More specifically, PLAL can be considered a “chemically green” procedure, that provides rather unique synthesis conditions such as high temperature and GPa pressure gradients. This permits the production of systems with unique morphology [27,28,29,30,31,32,33,34], structure, and composition, including rarely observed nano-alloys and nanomaterials containing meta-stable phases [20,35,36]. Simplicity of the methods, its scalability, and diversity of source materials for nanoparticles formation, as well as deepening insight into the physics of laser-matter interaction make PLAL synthesis attractive for multigram-scale production of inexpensive nanomaterials for various practical applications.

Enormous efforts were made so far to obtain nanomaterials made of pure noble metals [31,37,38,39] using PLAL, while only a few studies attempted to adopt this method for combining noble metals with common semiconductor—silicon—within unified hybrid nanostructrues. In particular, Au-Si hybrids with various morphologies (such as core–shell, core-satellites, nanoalloy, nanosponges, etc.) were reported by several research groups highlighting their unique optical and nonlinear optical properties as well as high potential for nanoscale light emission, SERS-based biosensing, light-to-heat conversion, and phototherapy [21,22,23,24,25,26]. Saraeva, et al. demonstrated general applicability of PLAL-synthesis to produce Si microspheres decorated by Ag nanoclusters [40], yet structural features of the produced nanomaterial, its optical properties, and related applications were left behind.

In this study, high-quality nanomaterial in the form of Ag-decorated Si microspheres was produced upon single-step nanosecond (ns)-laser ablation of monocrystalline silicon in isopropanol containing optimized content of AgNO_3_ precursor. At fixed laser irradiation time, salt content in the working solution was found to define the decoration degree with Ag nanoclusters that preferentially grow on the Si surface via thermal-induced decomposition of the precursor molecules. Morphology and inner structure of the as-prepared Ag-Si hybrids were comprehensively characterized by combining state-of-the-art 3D tomographic reconstruction with scanning electron microscopy (SEM) and focused ion-beam (FIB) milling, transmission electron microscopy (TEM) with energy-dispersive X-ray (EDX) chemical mapping modality as well as X-ray diffraction (XRD). Finally, our studies revealed applicability of the produced Ag-decorated microspheres as a versatile SERS platform offering temperature-feedback modality as well as single-particle signal enhancement that is well enough for fingerprint identification of the analyte molecules and tracing their plasmon-driven catalytic transformations.

## 2. Materials and Methods

### 2.1. PLAL Synthesis

Hybrid Ag-Si microspheres were produced via single-step laser ablation of monocrystalline Si wafer in isopropanol (purity grade > 99.9 wt.%, Sigma-Aldrich) containing silver nitrate (AgNO_3_, purity 99.9%, Sigma-Aldrich, St. Louis, MO, USA). Non-doped (>2000 Ω˖cm) Si wafer with (001) surface orientation was used in all laser ablation experiments. Prior to PLAL synthesis, Si target was subjected to a standard RCA cleaning procedure followed by a short dip in hydrofluoric acid and deionized water rinsing. In particular, Si wafer was fixed in a quartz cuvette containing a working solution made by mixing 9 mL of isopropanol and 1 mL of aqueous solution of AgNO_3_. To control the decoration degree of the product, the amount of the AgNO_3_ was varied from 5 × 10^−^^5^ to 10^−^^2^ M. Laser pulses (pulse duration of 7 ns, wavelength of 532 nm, and pulse repetition rate of 20 Hz) generated by Nd:YAG laser source (Ultra, Quantel, Lannion, France) at fixed pulse energy of ≈1.5 mJ were focused by a lens with a focal distance of 20 cm on the Si wafer surface. Such focusing parameters allow to ablate the circular-shape area (≈0.13 mm^2^) upon continuous single-spot irradiation. In all the experiments, laser exposure was carried out for 2 h upon continuous steering of the working solution.

### 2.2. Characterization

Morphology and inner structure of the Ag-Si hybrids were studied using SEM combined with a FIB milling modality (Helios 450s Nanolab, Thermofisher, Waltham, MA, USA). FIB milling of Pt-protected microspheres was carried out at 30 keV and a beam current of 25 pA to prepare their thin lamella for high-resolution TEM (HR-TEM) as well as to produce multiple slices for 3D tomographic reconstruction. The later procedure was performed with FEI AutoSlice & View G3 software (Thermofisher, USA), resulting in fabrication of about 110 slice images of the isolated nanostructure with an average slice thickness of 10 nm. The slice images were further processed with Avizo 8.1 software (Thermofisher, USA) to create the 3D model of the microsphere. Scanning TEM (STEM) imaging combined with an EDX spectroscopy (EDAX) for chemical mapping was carried out at an acceleration voltage of 300 kV (Titan 60–300, Thermo Fisher, USA).

To determine the phase composition and crystallinity of the Ag-Si microspheres supported by crystalline Si substrate, XRD method (Rigaku SmartLab, Tokyo, Japan) with Cu Kα radiation and parallel beam optics in 2θ/ω mode was used. The XRD peaks were identified using the ICSD database.

### 2.3. Applications

SERS experiments were carried out using commercial confocal laser system (Ntegra Spectra II, NT-MDT, Russia) equipped with CW laser sources (wavelengths λ_pump_ of 473 and 633 nm), focusing system with a dry microscope objective having numerical aperture (NA) of 0.7 (100×, M Plan Apo, Mitutoyo, Kawasaki, Japan), an avalanche photodetector for reflectivity mapping as well as an optical spectrometer with a monochromator (M522, Solar Laser Instruments, Belarus) and a thermo-electrically cooled CCD camera (i-Dus, Oxford Instruments, UK). An additional photodetector was used to control the laser intensity at the output of the focusing objective. The focal depth estimated as ≈ λ_pump_/NA^2^ ≈ 0.96 μm (at λ_pump_ ≈ 473 nm) ensured uniform excitation of the microspheres on smooth substrates. Lateral size of the focal spot (≈ 1.22λ_pump_/NA) was about 0.8 and 1.1 μm at λ_pump_ = 473 and 633 nm, respectively, also matching the Au-Si microsphere size. Prior to SERS measurements, Ag-Si microspheres were maintained in the isopropanol solutions containing Raman-active molecules, Rhodamine 6G (R6G), and para-aminothiophenol (PATP), at their molar concentrations of 10**^−^**^6^ M for 1 h. Then, the microspheres were cleaned with distilled water several times and drop-casted onto a smooth Ag mirror. Such mirrors were produced by covering monocrystalline Si wafer with a 500 nm thick silver film using magnetron sputtering. The concentration of the PLAL-synthesized nanomaterial in the distilled water was chosen to provide separated microspheres on the surface upon drying. All Raman and SERS measurements were performed on isolated Ag-Si microspheres.

Finite-difference time-domain (FDTD) simulations were undertaken with a commercial electromagnetic solver (Lumerical Solutions, Ansys, Canonsburg, PA, USA) to calculate the local structure of the electromagnetic fields in the vicinity of Ag-Si microspheres upon their excitation with various pump wavelengths ranging from 405 to 633 nm. The dielectric constants of silver and silicon at corresponding wavelength were taken from [41]. Simulations were carried out considering isolated Ag-Si microsphere with the precise geometrical model taken from experimental 3D tomographic reconstruction. Microsphere was irradiated from the top by linearly polarized plane wave. Simulation volume was limited by perfectly matched layers, while the size of the elementary cell was fixed at 1 × 1 × 1 nm^3^.

## 3. Results and Discussions

### 3.1. PLAL Synthesis of the Ag-Si Hybrids and Their Characterization

**Figure 1:**
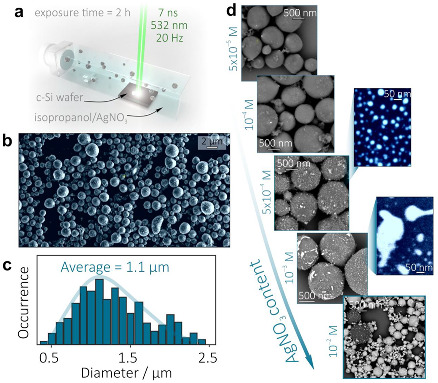
(**a**) Schematic of the PLAL synthesis of Ag-Si microspheres upon ns-laser ablation of the monocrystalline Si wafer placed in the isopropanol containing AgNO_3_. (**b**) Top-view SEM image of the as-synthesized Ag-Si product dried on a Si wafer. (**c**) Size distribution of the Ag-Si microspheres. (**d**) Series of SEM images illustrating evolution of the density and geometry of the Ag nanoclusters on the surface of Si microspheres upon increase of the AgNO_3_ content in the working solution from 5 × 10^−5^ to 10^−2^ M.

Morphology of the nanomaterial produced upon direct ns-laser ablation of monocrystalline Si wafer in the working solution containing isopropanol and AgNO_3_ (5 × 10^−4^ M) for 2 h is illustrated in Figure 1b. PLAL-synthesized hybrids drop-casted onto the Si wafer generally represent spherical-shape Si particles decorated with isolated or merged Ag nano-clusters. Statistical analysis of the multiple SEM images gave an average diameter of the Ag-Si microspheres of ≈1.1 µm with a rather broad distribution of their size ranging from 0.7 to 1.6 µm (according to the FWHM of the Gaussian fit; Figure 1c).

Taking into account rather long 7-ns laser pulses used in the experiments, the Ag nanoclusters are expected to be produced upon thermal-induced decomposition of the AgNO_3_ to metallic silver phase [42,43]. Once the working solution is completely transparent at 532 nm wavelength laser radiation, the photo-induced AgNO_3_ decomposition is expected to be much less efficient for the chosen laser fluence and low concentration of the AgNO_3_ (<10^−3^ M), while no presence of the pure Ag nanoparticles was found for similar experiments performed upon irradiation of the working solution without Si wafer. Meanwhile, as the Si particles are formed and ejected into the working solution upon PLAL synthesis, these particles further act as a local laser-radiation absorbers and nano-heaters that give surface sites for preferential growth of the Ag nanoclusters. For the fixed PLAL parameters (laser wavelength, pulse duration, fluence, and irradiation time), the initial amount of the AgNO_3_ in the working solution allows control over the amount and the geometrical shape of the formed Ag nanoclusters. More specifically, the surface density of these metallic nanoclusters gradually increases at AgNO_3_ concentrations ranging from 10^−5^ to 10^−2^ M (Figure 1d). The separated nanoclusters typically exhibit regular shapes, according to top-view SEM images and the size ranging from 10 to 30 nm (middle panel, Figure 1d). As the density of the nanoclusters increases, they can form dimers and more complex-shape arrangements of the Si microsphere surface that are preferential for light localization and enhancement within broad spectral range. Meanwhile, at higher AgNO_3_ concentrations (10^−3^ M), excessive formation of the Ag nanoclusters leads to their merging into larger irregular-shaped agglomerates upon laser-induced remelting. Finally, even larger content of AgNO_3_ stimulates enormous growth and merging of Ag nanoparticles in the working solution without any preferential growth. The resulting nanomaterial mainly contained pure Ag nanoparticles with their size reaching up to 500 nm, while small amounts of Ag-Si hybrids arr observed (bottom panel, Figure 1d). Related EDX studies of the Ag-Si nanopowders produced at AgNO_3_ concentration ranging from 10^−4^ to 10^−3^ M (that provided formation of the Ag-decorated Si microspheres) showed increase of the average Ag content from 2 to 13 wt.%.

**Figure 2:**
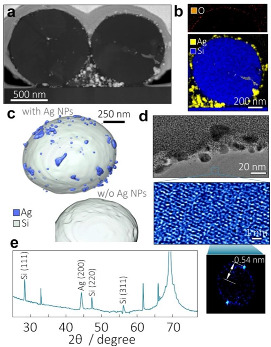
(**a**) STEM image of the cross-sectional central cut made through the 1.1-µm diameter Ag-Si microspheres; (**b**) EDX composition mapping of the right-most Ag-Si microsphere showing distribution of Ag and Si elements as well as an oxide shell (top panel); (**c**) 3D model of the isolated Ag-Si microsphere made through SEM tomographic reconstruction from the series of multiple images of cross-sectional cuts. Bottom panel shows the same model where Ag nanoclusters were removed to illustrate crater-like Si surface morphology; (**d**) TEM image of the Ag-Si microsphere surface (top panel) as well as HR-TEM image and its FFT showing crystalline structure of the Si core (bottom panel); (**e**) XRD pattern of the Ag-Si nanopowder. All unmarked peaks represent contribution from the underlying Si substrate.

Deeper insight into morphology, structure, and composition of the Ag-Si hybrids produced at AgNO_3_ content of 5 × 10^−4^ M was provided by combining advanced TEM imaging modalities including 3D tomographic reconstruction and high-resolution EDX elemental mapping. Figure 2a provides the STEM image of one representative cross-sectional FIB cut made through the geometric center of two neighboring 1.1-µm diameter Ag-Si hybrids. EDX analysis shows that negligibly small amount of Ag can be observed inside the bulk of the Si microsphere (bottom panel, Figure 2b). Few nm thick oxide shell was found to wrap the Si microsphere, indicating week oxidation process of the silicon upon PLAL synthesis (top panel, Figure 2b). EDX mapping also indicates presence of carbon species (≈3–5 wt.%) evidently coming from decomposition of the isopropanol molecules. Appearance of pure Ag colloid agglomerating in the free space between two Ag-Si microspheres and the Si wafer can indicate non-preferential growth of the Ag nanoparticles in the working solution and/or their laser-induced removal from the Si microsphere surface by subsequent laser pulses. Further optimization of the PLAL synthesis parameters (exposure time and fluence) are to be carried out to reduce or completely remove (if required) the free Ag colloid from the resulting product. Meanwhile, such optimization goes beyond the scope of this work.

3D tomographic model created by post-processing of multiple SEM images of the FIB cross-sectional cuts is shown in Figure 2c, providing visual perception of the Ag-Si microsphere morphology and composition. This data also suggests that certain Ag nanoclusters (both isolated and merged) are partially embedded to the Si microsphere surface (bottom panel, Figure 2c), while the Si core surface has a crater-like morphology.

The same justification can be made upon analyzing close-up TEM images of the Ag-Si microsphere surface shown in Figure 2d. HR-TEM imaging also confirms the crystalline structure of the Si core (bottom panel, Figure 2d) and its lattice constant ≈0.54 nm according to the Fast Fourier Transform analysis. XRD studies of the Ag-Si nanopowder finalize comprehensive structural and compositional characterization of the produced nanomaterial. The sharp diffraction peak at 2Θ = 28.36° (see Figure 2e) corresponding to the diffraction from Si(111) planes together with less intensive peaks at 47.3° and 56.1° originate from that of Si(220) and Si(311), respectively. Despite sub-micron diameter of the obtained Ag-Si microspheres, they demonstrate rather high crystallinity with a calculated mean size of the crystallite ~180 nm in accordance with Scherrer’s equation. Ag contribution is presented by a diffraction peak at 44.4° with its FWHM of 0.31°, which provides Ag crystallites mean size to be 28 nm. No signal features related to Ag silicides (either Ag_2_Si or Ag_3_Si) [44,45] were observed in the XRD patterns. However, related Ag(200) peak is shifted towards smaller 2Θ (larger Ag lattice constant) compared to those for bulk Ag, which can be explained by certain Ag-Si alloying upon laser ablation.

### 3.2. SERS Performance of the Ag-Si Hybrids

**Figure 3:**
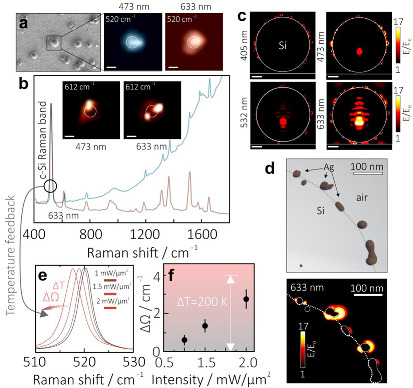
(**a**) Reference SEM image of the Ag-Si microspheres drop-casted onto the Si wafer as well as Raman images (at 520 ± 2 cm^−1^) of the chosen isolated nanoparticle at 473 and 633 nm laser pump wavelengths. (**b**) Representative averaged SERS spectra of the R6G capping the same Ag-Si microsphere at 473 and 633 nm laser pump wavelengths. Top insets show the distribution of the SERS signal (at 612 ± 2 cm^−1^) near the microsphere. (**c**) Normalized electric-field amplitude E/E_0_ near the isolated Ag-Si microsphere on the Ag mirror upon its excitation with a linearly polarized plane wave at 405, 473, 532, and 633 nm. (**d**) Close-up distribution of E/E_0_ near the isolated Ag nanoclusters on the Si surface at 633 nm pump as well as corresponding fragment of the 3D tomographic model of the Ag-Si microsphere used for modeling. (**e**) Shift of the detected c-Si Raman band at 520 cm^−1^ upon increasing the laser pump intensity (633 nm) of the Ag-Si microsphere from 0.5 to 2 mW/µm^2^. (**f**) Thermally induced shift of the c-Si band ΔΩ as a function of laser pump intensity.

As it was mentioned in the introduction section, nanostructures made of plasmonic metals and semiconductors represent promising platform for reliable optical nanosensing based on the SERS effect. To justify the applicability of the PLAL-synthesized Ag-Si microspheres (produced at AgNO_3_ concentrations of 5 × 10^−4^ M), we first functionalized them with R6G molecules (10^−6^ M alcohol solution) representing a common molecular probe that exhibits good affinity to noble metals and high Raman yield. The R6G-capped hybrids were then drop-casted onto a smooth Ag mirror in such a way to form the isolated structures on the surface that was confirmed by SEM imaging (Figure 3a; left panel). As can be seen, hybrids can also agglomerate to form a dimers or more complicated surface arrangements. However, to quantitatively assess SERS performance of PLAL-synthesized hybrids we restricted out study by the case of isolated Ag-Si microspheres.

Inherent Raman scattering of Si core (Raman band at ≈520 cm^−1^) allows the exact position of Ag-Si microspheres on the surface to be easily found, aligns them with respect to the pump laser source, and relates their spatial position with the detected SERS signal from the analyte molecules. The Raman image (520 cm^−1^) of a representative 1-µm diameter Ag-Si microsphere measured using 473 and 633 nm pump wavelengths are provided in Figure 3a. The images highlight the ability of the setup to easily resolve the isolated hybrids, Raman mapping resolution ≈ 1 µm and a uniform distribution of the signal with its maximum matching the center of the Ag-Si microsphere. For both pump wavelengths, we also plotted the R6G SERS images (characteristic band at 612 cm^−1^; insets of Figure 3b) showing rather random signal distributions for different wavelengths within the microsphere area highlighted by the white ring. R6G SERS spectra averaged over the integral signal coming from the nanostructure (highlighted by white ring) are also plotted in Figure 3b, clearly showing main characteristic bands of the analyte molecules, yet with a strong contribution of the analyte photoluminescence upon its excitation at 473 nm. By comparing the averaged R6G SERS yield (total intensity of the bands at 612 and 1652 cm^−1^) coming from the microsphere area (≈3.92 × 10^4^ and 5.71 × 10^4^ cps per 1 mW/µm^2^ for 473 and 633 nm pump wavelength, respectively) with the same signal from the R6G-capped smooth Ag mirror (4–10 cps per 1 mW/µm^2^ for both wavelength), we estimated the electromagnetic contribution to the SERS enhancement factor to be at least ~10^4^ for single Ag-Si microsphere. Considering additional chemical contributions coming from the interaction of the Raman probe with plasmon metal, the total SERS enhancement factor reaches 10^6^ and is expected to be even larger for agglomerated microspheres.

Interestingly, the R6G SERS yield at the 633 nm pump wavelength is evidently higher than it is counter-intuitive, considering that the main contribution is expected to come from Ag nanoclusters that should exhibit stronger plasmonic response at shorter wavelengths. To clarify this feature, we carried out FDTD simulations of the local EM-field structure near the Ag-Si microsphere surface at various pump wavelengths ranging from 405 to 633 nm. 3D model of the microsphere for simulations was elaborated using the data from tomographic SEM reconstruction (Figure 2c). The main results of these simulations are summarized in Figure 3c, revealing several interesting observations. First of all, the main EM “hot spots” are expectedly related to the Ag nanoclusters, while the most intense normalized EM-field amplitude (E/E_0_) is observed at 633 nm pump providing electromagnetic SERS enhancement factor~(E/E_0_)^4^ up to 10^5^. Indeed, the Ag nanoclusters with the size comparable to the double skin depth should demonstrate plasmonic response in the UV spectral range. However, these clusters are strongly attached or partially embedded into the Si core, as was shown by the microscopic analysis (Figure 2c,d).

Strong interaction of the plasmonic nanoparticles with high-refractive-index Si usually causes redshift and hybridization of localized plasmon resonances. Irregular elongated shape of the Ag nanoclusters and inter-particle interaction also reasonably explain redshift of the plasmonic resonances (Figure 3d). Meanwhile, the main reason that the high SERS yield at 633 nm pump is related to gradually increasing transparency of the Si material in the red part of the spectrum. This feature allows the Si core to act as an efficient Fabry–Perot cavity upon pumping from the top by the plane wave, which leads to multiple excitation cycles of the interface Ag nanoclusters by the radiation bounded within the Si core. Localization of the EM field inside the Si core as well as standing-wave-like amplitude modulations confirms this deduction. Transparent Si core also allows the pumping of the Ag nanoclusters located in the bottom hemisphere that cannot be pumped by the laser radiation incident from the top in the case of the opaque core (for example, at 405 nm pump).

Apart from the convenient ability to identify spatial position of the Ag-Si microspheres, characteristic c-Si Raman band also allows to trace the local temperature in the analyte-nanoparticle system. Analytes for SERS experiments usually represent organic or biological species with rather low thermal decomposition temperatures (i.e., R6G molecules decompose slightly above 500 K [46]). Thus, it is important to maintain the temperature at a reasonable level during SERS experiment. Upon laser-induced heating, the maximum of the c-Si Raman band blue-shifts, as it is illustrated by representative series of Raman spectra measured from the isolated Ag-Si microsphere, (produced at AgNO_3_ concentrations of 5 × 10^−4^ M) pumped at various laser intensity up to 2 mW/µm^2^ (Figure 3e).

**Figure 4:**
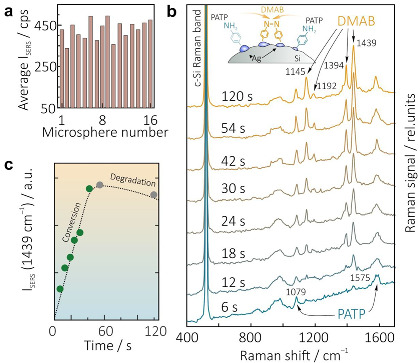
(**a**) R6G SERS yield (intensity of the bands at 612 and 1652 cm^−1^) statistically averaged over measurements from 16 randomly chosen isolated Ag-Si microspheres produced at AgNO_3_ concentrations of 5 × 10^−4^ M. (**b**) Series of time-resolved SERS spectra of the PATP capping the isolated Ag-Si microsphere. Total laser exposure time ranging from 6 to 120 s is indicated near each spectrum. (**c**) Evolution of the intensity of the characteristics DMAB-related Raman band at 1439 cm^−1^ as a function of laser exposure time indicating PATP-to-DMAB conversion dynamics.

The spectral shift Δ*Ω* can be then recalculated to local temperature Δ*T* increase using the following formula [47]:

(1) $$∆\Omega =A\left(1+\frac{2}{e^{\frac{\hbar \Omega_{0}}{2kT}}-1}\right)+B\left(1+\frac{3}{e^{\frac{\hbar \Omega_{0}}{3kT}}-1}+\frac{3}{{\left(e^{\frac{\hbar \Omega_{0}}{2kT}}-1\right)}^{2}}\right)$$

where *Ω*_0_ = 528 cm^−1^, *A* = −2.96 cm ^−1^, *B* = −0.174 cm^−1^, *ħ* = 1.054·10^−34^ J·s, *k* = 1.38·10^−23^ J/K. Results of systematic measurement of the heating efficiency of the isolated Ag-Si microspheres of nearly similar diameter (1.1 ± 0.15 µm) at 633 nm pump are summarized in Figure 3e, showing that pump intensity of 2 mW/µm^2^ typically causes temperature ΔT increase up to ≈200 K. Importantly, local single-nanoparticle thermometry with an accuracy of ±20 K and high spatial resolution can be realized in parallel with a SERS identification of the analytes, allowing more reliable and stable measurements [48]. For laser pump intensity slightly below 2 mW/µm^2^, the Ag-Si microspheres produced at AgNO_3_ concentrations of 5 × 10^−4^ M demonstrate good reproducibility of the R6G SERS yield according to the systematic measurements performed for various microspheres of nearly similar size (Figure 4a). Noteworthy, we also carried out systematic studies of the SERS yield, its reproducibility, and heating efficiency provided by the isolated Ag-Si microspheres PLAL-synthesized at larger (10^−3^ M) amount of AgNO_3_. Despite evidently larger average SERS yield related to R6G probe, such microspheres also exhibit substantially larger deviation of the both SERS enhancement and heating efficiency limiting their application for reliable and precise measurements.

Strong and reliable SERS enhancement combined with local thermometry make Ag-Si hybrids promising for realization of advanced nanosensing platforms. To further support this statement, we applied them for SERS-based detection of plasmon-induced catalytic transformation of PATP to dimercaptoazobenzene (DMAB). Similarly to previously described experiments with R6G, Ag-Si microspheres were functionalized with PATP molecules (5 × 10^−6^ M, methanol solution) and drop-casted onto the Ag mirror substrate to form the isolated structures. SERS spectrum of pristine analyte contains only two Raman bands centered at 1079 and 1575 cm^−1^, while upon its catalytic dimerization to DMAB four additional lines appear in the spectrum at 1145, 1192, 1394, and 1439 cm^−1^ [10,49,50]. According to thermogravimetric analysis, decomposition temperature of PATP is ≈450 K [51], thus pump intensity was set slight below 1.5 mW/µm^2^ to avoid analyte degradation. Representative series of SERS spectra measured by increasing the laser exposure time of PATP-capped Ag-Si microsphere from 6 to 120 s is provided in Figure 4b showing appearance and gradual increase of the intensity of all DMAB-related Raman bands. Meanwhile, after 60 s of continuous laser exposure, the intensity of these bands starts to decay revealing complete dimerization of pristine molecules to the product and start of the photodegradation process. Mentioned dynamics is illustrated by Figure 4c, where the intensity of the brightest DMAB band at 1439 cm^−1^ (that is typically used to assess PATP-to-DMAB conversion dynamics) is plotted versus the overall laser exposure time. As can be seen, properly chosen (thermally safe) pump intensity allows the tracing of the mentioned conversion processes in detail within a reasonable time and at good SERS signal level revealing applicability of the designed single-particle platform for studying the molecular transformations under some external stimuli.

## 4. Conclusions

To conclude, we demonstrated one-step/one-pot synthesis of Ag-decorated Si microspheres via ns-laser ablation of monocrystalline silicon in isopropanol containing AgNO_3_. Once the ejected Si microspheres strongly absorb subsequently incident laser pulses, the preferential thermal reduction of AgNO_3_ to metallic silver phase on the laser-heated Si microsphere surface reasonably explains their decoration process. Demonstrated submicron-scale hybrid Ag-Si particles are promising for temperature-feedback SERS bio- and chemo-sensing, while the size of the isolated microsphere ideally matches the focal spot size of the pump laser radiation, allowing reliable single-particle measurements at substantially large enhancement factor of 10^6^. At the same time, the size of the Si core in the resulting Ag-Si hybrids is defined by the averaged size of the nanoparticles ejected upon ns-laser PLAL processing of the silicon surface. This means that the processing parameters can be optimized in such a way to obtain smaller averaged size of the Si nanoparticles in the suspension and thus reduce the average size of Ag-Si hybrids. In its turn, this can be achieved by decreasing the pulse duration, tuning laser wavelength that defines penetration depth of the radiation to the material, as well as by varying the type of the substrate (for example, by using Si nanowires or porous Si [52]).

## Data Availability

Data will be made available on request.
